# Priority-setting dilemmas, moral distress and support experienced by nurses and physicians in the early phase of the COVID-19 pandemic in Norway

**DOI:** 10.1177/0969733020981748

**Published:** 2021-01-12

**Authors:** Ingrid Miljeteig, Ingeborg Forthun, Karl Ove Hufthammer, Inger Elise Engelund, Elisabeth Schanche, Margrethe Schaufel, Kristine Husøy Onarheim

**Affiliations:** 60518 University of Bergen, Norway; 60498 Haukeland University Hospital, Norway; 60518 University of Bergen, Norway; 60498 Haukeland University Hospital, Norway; 4919 University College London, UK; University of Bergen, Norway

**Keywords:** Clinical ethics, ethics and leadership/management, moral distress, moral/ethical climate of organisations, organisational ethics, professional ethics

## Abstract

**Background:**

The global COVID-19 pandemic has imposed challenges on healthcare systems and professionals worldwide and introduced a ´maelstrom´ of ethical dilemmas. How ethically demanding situations are handled affects employees’ moral stress and job satisfaction.

**Aim:**

Describe priority-setting dilemmas, moral distress and support experienced by nurses and physicians across medical specialties in the early phase of the COVID-19 pandemic in Western Norway.

**Research design:**

A cross-sectional hospital-based survey was conducted from 23 April to 11 May 2020.

**Ethical considerations:**

Ethical approval granted by the Regional Research Ethics Committee in Western Norway (131421).

**Findings:**

Among the 1606 respondents, 67% had experienced priority-setting dilemmas the previous two weeks. Healthcare workers who were directly involved in COVID-19 care, were redeployed or worked in psychiatry/addiction medicine experienced it more often. Although 59% of the respondents had seen adverse consequences due to resource scarcity, severe consequences were rare. Moral distress levels were generally low (2.9 on a 0–10 scale), but higher in selected groups (redeployed, managers and working in psychiatry/addiction medicine). Backing from existing collegial and managerial structures and routines, such as discussions with colleagues and receiving updates and information from managers that listened and acted upon feedback, were found more helpful than external support mechanisms. Priority-setting guidelines were also helpful.

**Discussion:**

By including all medical specialties, nurses and physicians, and various institutions, the study provides information on how the COVID-19 mitigation also influenced those not directly involved in the COVID-19 treatment of patients. In the next stages of the pandemic response, support for healthcare professionals directly involved in outbreak-affected patients, those redeployed or those most impacted by mitigation strategies must be a priority.

**Conclusion:**

Empirical research of healthcare workers experiences under a pandemic are important to identify groups at risks and useful support mechanisms.

## Introduction

During outbreaks of new diseases such as COVID-19, ethically demanding situations arise.^1–3^ Priority-setting dilemmas emerge due to shortage of both medical and human resources.^4,5^ Diagnostics, treatment and care that is normally provided and considered useful might be delayed or restricted to redistribute scarce resources in response to the pandemic.^6,7^ Employees with one set of skills may also be redeployed and expected to perform tasks that require skills in which they are less experienced. When resources are limited, clinicians face challenging priority-setting dilemmas when having to choose whom to admit or to give the recommended treatment.^8,9^

How ethically demanding situations are handled has been shown to impact employees’ moral distress and job satisfaction.^10,11^ A common definition of moral distress is stress or worry that health professionals may experience when various obstacles or barriers prevent them from performing what they believe is ethically correct.^12^ The extent of perceived moral distress is influenced by a variety of factors, including personal attributes, ward and hospital conditions and external factors, such as an pandemic.^13–15^

During pandemics or disasters, healthcare workers may be particularly exposed to priority setting and other ethical dilemmas caused by scarcity of resources and might experience high levels of moral distress.^16–18^ Empirical research indicates that identifying and responding to staff who are experiencing ethical dilemmas in pandemics is important to reduce adverse consequences for patients, moral distress among healthcare workers and poor work environments.^17,19^

Norway was hit by the COVID-19 pandemic in early March 2020.^20^ Following a rapid increase of new cases, and influenced by the dramatic and unpredictable situation in Italy and other severely affected countries, the government’s response in mid-March led to a ‘shutdown’. This national shutdown included both drastic changes within the healthcare system and non-health interventions, such as school and kindergarten closure, advice against non-essential travel and working-from-home recommendations.

The Norwegian healthcare system is publicly financed, and specialised healthcare services are provided free of charge. Initial estimates in March 2020 indicated a potential peak of infections in April–May. These estimates led health trusts to implement drastic measures, changing everyday routines from 1 week to the next.^20,21^ Pandemic response plans were made, and wards and hospitals were reorganised to mitigate the expected rapid increase in numbers of COVID-19 patients. Additional equipment was purchased and existing resources were reallocated, including cancelling and scaling-down elective surgery and other non-emergency services or delivering outpatient services online.^20^ Healthcare workers were redeployed to other wards or departments and provided COVID-19 specific training. For instance, in the Helse Bergen health trust, most elective orthopaedic surgeries were postponed, and the nurses in the orthopaedic ward were trained to be redeployed to the pandemic ward or other wards in need of staff. COVID-19 guidelines and response plans were developed at the national and local level.^22^ Guidelines included criteria for priority setting and triage, procedures and regulation regarding visitors, palliation, collaboration with primary healthcare institutions and sub-speciality guidance.^23^ Overall, these changes had substantial effects on the management of both COVID-19 and other patients, as well as the everyday practice of healthcare professionals in Norway.^24^

The unprecedented and rapidly implemented changes COVID-19 has imposed on healthcare delivery, managers and frontline workers worldwide require further scrutiny. The experiences from the early phases of the pandemic should be used to prepare for the next stages of the pandemic and in responses to future public health emergencies. The aim of this study is thus to describe priority-setting dilemmas, moral distress and support experienced by nurses and physicians in the early phase of the COVID-19 pandemic in the Western part of Norway.

## Methods

The study is a cross-sectional, hospital-based survey conducted in the western health region of Norway from 23 April to 11 May 2020.

### Study context

The survey was conducted after the initial stabilisation of the number of COVID-19 infections and deaths, but changes implemented in the hospitals were still in place.^25^ The Western health region (Helse Vest) covers a population of 1.1 million citizens and an area of 43,000 square kilometres (twice the area of Wales). The yearly budget is 30 billion NOK (2.9 billion Euros) and the region consists of five local health trusts, including 50 different institutions providing specialised healthcare at university hospitals, regional hospitals, local hospitals and various other units. Respondents from four of five local health trusts in Helse Vest were included in the study (Helse Bergen, Helse Stavanger, Helse Førde, Haraldsplass Deaconess Hospital). The fifth local health trust, Helse Fonna (the smallest one), abstained from participating due to limited capacity. The health trusts vary in size, catchment area and capacity to handle various kinds of patient groups. Before COVID-19, the ICU capacity was two ICU beds in Helse Førde and in Haraldsplass Deaconess Hospital, 8 in Helse Stavanger and 20 in Helse Bergen. All of the trusts had more ICU beds available, but these are used in high-dependency units, post-anaesthesia care units, and so on.^i^

From the initial lockdown to the end of the study, the total caseload of COVID-19 patients included 159 admissions in the region, out of which 31 received treatment in intensive care units (ICUs). Ten COVID-19-related hospital deaths had been reported by 11 May 2020. The numbers of COVID-19-patients in the local health trusts varied; Haraldsplass Deaconess Hospital (HDS) received a relatively higher number of cases (31) compared to the number of hospital beds. Only a few cases (8) were treated in Helse Førde. Due to the expected wave of infections and burden on the health care system, everyday routines were changed, and responses were implemented in all local health trust.

All nurses and physicians employed in a ≥ 20% position and all managers involved in clinical work in the health trusts received personal e-mail invitations to participate in the study (two reminders). All hospital employees have a work e-mail address, and the human resources department in each health trust extracted lists of employed nurses, physicians and managers meeting the inclusion criteria. The e-mail invitation to potential participants included information about the study, a letter of informed consent and a personal electronic link to the online survey.

### Survey instrument

The survey included questions on demographics, occupational background and three main themes: priority-setting dilemmas, moral distress and employee support. Questions on clinical priority-setting dilemmas and resource scarcity were developed based on existing literature and adapted to a Norwegian hospital setting.^8,9^ Respondents were asked how often they had experienced ethical dilemmas related to resource constraints (referred to as *priority-setting dilemmas* in this paper) and the most adverse consequence of resource scarcity they had seen the previous 2 weeks. Resource constraints were described as ‘for instance lack of material resources (personal protection equipment, beds, ventilators, etc.) or human resources (not enough people at work, not enough qualified personnel, etc.)’. Possible consequences for patients were: no consequences; modified or no treatment, but without serious consequences; accidents without serious consequences; temporary loss of function; permanent loss of function; an acute life-threatening event; death.

The Moral Distress Thermometer (MDT), a tool used across professions and settings^26–28^ was used as a general, rapid and easy-to-understand measure of moral distress. In the survey, moral distress was defined as: ‘Studies show that health care providers can experience what is called moral distress. Moral distress occurs when you believe you know the ethically correct thing to do, but something or someone restricts your ability to pursue the right course of action’. Respondents were asked to indicate their level of moral distress on a scale from 0 (‘None’) to 10 (‘Worst possible’). The MDT was translated into Norwegian and adapted to the online survey software. The visual layout of the MDT scale therefore differed slightly from the original MDT.

Questions on perceived support included issues shown to be important for health professionals during pandemics and for preventing moral distress and burnout.^16–18^ Respondents were asked to which degree they had experienced a specific type of support as helpful. The survey was designed to be brief (10–15 minutes) in order to respect the time-pressure on the healthcare workers. The survey (paper and online versions) was piloted and pre-tested by eight clinicians to increase reliability and validity.^29^

### Statistical analysis

For exploring the relationship between various demographics and occupational factors and the frequency of experiencing priority-setting dilemmas and of the level of moral distress, we fitted multivariable regression models – proportional odds (cumulative logit) and linear regression models, respectively. The proportional odds model was also used for all other ordinal response variables. We aimed to predict the frequency of experiencing priority-setting dilemmas and the level of moral distress on an individual level, so all demographic and occupational factors were included in the multivariable analyses.

We hypothesised that the effect of belonging to a specific department or hospital on the above outcome variables might vary according to whether the health worker was directly involved with COVID-19 patients or redeployed. For example, the differences across departments might be expected to be larger for those health workers directly involved if departments varied in their preparedness to the pandemic. To explore this, we ran additional models where we included an interaction term between department or hospital and being directly involved or redeployed. Furthermore, we hypothesised that the predicted effect of having managerial responsibilities would vary according to direct involvement or redeployment. Therefore, we ran models where we included an interaction term between being a manager and being directly involved or redeployed. No other interaction tests were performed. Interactions between variables were tested using likelihood ratio tests to compare models with and without an interaction term.

For presenting the frequency of priority-setting dilemmas for various subgroups, we scored the responses: ‘never’ as 0, ‘fewer than weekly’ as 1, ‘weekly’ as 2, ‘several times a week’ as 5, ‘daily or almost daily’ as 10 and ‘several times a day’ as 20. This roughly corresponds to the number of priority-setting dilemmas experienced in the previous 2 weeks. We compared means across groups using Welch’s ANOVA or Welch’s *t*-test.

All questions in the electronic survey were ‘required’, so there are no missing data. For the questions with response options ‘Don’t know’ or ‘Other’, we report the number of each type of response.

All analyses were conducted using Stata version SE 16.1 or R version 4.0.0.^30^ *P*-values ≤ 0.05 are characterised as statistically significant.

## Ethical considerations

Ethical approval granted by the Regional Research Ethics Committee in Western Norway (131421). The study was planned in the early beginning of the pandemic (March 2020), when it was very uncertain how the pandemic and lockdown measures would hit Norway and the health care system. The research group established an advisory board which consisted of hospital trusts senior advisers, safety and human resources representatives, one patient representative, one representative of staff. The advisory board provided guidance on timing, permission and ways of reaching potential participants, concerns for overburdened health care workers and called for rapid analysis to inform strategies for psychosocial follow-up of their employees.

Information about the study was available on internal webpages in each hospital trusts, on social media, physical posters in the wards and by direct communication with the directors at department level. Information about the study, anonymity, voluntariness and options to withdraw was provided in the e-mail to participate in the study and in the letter of informed consent. It was not possible to participate in the survey without indicating that information about the study had been provided and understood and that the participant wished to take part in the study.

Potential harms of the study included employee time to participation. To minimise the harms for patients and staff, the survey piloted and estimated to take10-15 minutes. While there were no direct harms of participation, participating in the study may activate frustration, moral distress, uncertainty and lack of control. The e-mail invitation sent to potential participants the letter of informed consent and the questionnaire itself provided information on name, email and phone number on whom to contact if they felt distressed or needed someone to talk to. A project webpage also included this information and provided a platform to pose questions to the researchers.

## Results

There were 1,748 respondents of the 9,702 who were invited (response rate: 18%), but 142 individuals were excluded as they were not involved in clinical work. The results presented are based on 1,606 responders, 67% and 31% of them identified as nurses and physicians, respectively (Table 1). In all, 37% of the respondents had been directly involved in treatment or care for COVID-19-patients, and 21% reported that they had been redeployed as part of the COVID-19 response.

**Table 1.** Demographic and occupational characteristics of respondents (*n* = 1606).

Characteristic	Count	Prop.(%)
Age		
20–29 years	228	14
30–39 years	432	27
40–49 years	485	30
50–59 years	323	20
60+ years	138	9
**Gender**		
Female	1235	77
Male	371	23
Other	0	0
**Health trust/ hospital**		
Helse Bergen	904	56
Helse Stavanger	489	30
Helse Førde	120	7
Haraldsplass Deaconess Hospital	93	6
**Time employed**		
< 1 year	147	9
(1–4) years	251	16
(4–10) years	350	22
(10–20) years	467	29
20 + years	391	24
**Main position**		
Specialist nurse	551	34
Nurse	525	33
Consultant	331	21
Registrar^a^	148	9
Junior doctor^b^	22	1
Other^c^	29	2
**Department**		
Medical specialities	686	43
Surgical specialities	364	23
Anaesthesia or intensive care medicine	251	16
Psychiatry or addiction medicine	234	15
Other^d^	71	4
**Manager**		
No	1375	86
Yes	231	14
**Directly involved in treatment** **or care of COVID-19 patients**		
No	1012	63
Yes	594	37
**Redeployed or given new** **responsibilities due to COVID-19**		
No	1276	79
Yes	330	21

^a^ Registrar includes doctors in specialisation (‘lege i spesialisering 2 og 3’).

^b^ Junior doctors includes interns and medical students with temporary licence to work as physicians.

^c^ Other specialities were responders only reporting ‘manager’ and other specific categorization.

^d^ Other departments included departments of radiology, laboratory medicine, pathology and departments where both medical and surgical patients were treated (in smaller units).

### Priority-setting dilemmas

Overall, 67% had experienced priority-setting dilemmas due to resource scarcity at least once in the previous two weeks. The mean score for priority-setting dilemmas was 1.8 (95% CI: 1.7–2.0). Differences across groups in how often the respondents had experienced the dilemmas are presented in Table 2.

**Table 2.** Mean scores for frequency of experienced priority-setting dilemmas and level of moral distress in the previous two weeks, stratified by demographic and occupational characteristics (*n* = 1,606).

	Ethical dilemmas^a^		Moral distress^b^	
	Mean (95% CI)	*p*-value	Mean (95% CI)	*p*-value
**Age**		0.21		0.14
20–29 years	2.1 (1.6–2.5)		3.2 (2.9–3.5)	
30–39 years	1.6 (1.4–1.8)		2.9 (2.7–3.1)	
40–49 years	2.0 (1.7–2.3)		2.9 (2.7–3.1)	
50–59 years	1.9 (1.5–2.2)		2.7 (2.5–3.0)	
60+ years	1.6 (1.1–2.2)		2.6 (2.2–3.0)	
**Gender**		0.75		0.002
Female	1.8 (1.7–2.0)		3.0 (2.9–3.1)	
Male	1.9 (1.6–2.2)		2.6 (2.4–2.8)	
**Time employed**		0.14		0.04
< 1 year	1.5 (1.1–2.0)		3.0 (2.6–3.4)	
(1–4) years	2.1 (1.7–2.5)		3.1 (2.9–3.4)	
(4–10) years	1.9 (1.5–2.2)		3.0 (2.7–3.2)	
(10–20) years	2.0 (1.7–2.3)		2.9 (2.7–3.1)	
20 + years	1.6 (1.3–1.9)		2.6 (2.4–2.8)	
**Health trust/hospital**		<0.001		<0.001
Helse Bergen	1.6 (1.4–1.8)		2.7 (2.5–2.8)	
Helse Stavanger	2.0 (1.7–2.3)		3.1 (2.9–3.3)	
Helse Førde	1.7 (1.2–2.3)		2.9 (2.5–3.4)	
Haraldsplass Deaconess Hospital	3.7 (2.6–4.8)		3.9 (3.4–4.4)	
**Main position**		0.20		0.007
Specialist nurse	2.0 (1.7–2.2)		3.0 (2.8–3.2)	
Nurse	1.8 (1.6–2.1)		3.1 (2.8–3.3)	
Consultant	1.9 (1.6–2.3)		2.6 (2.4–2.9)	
Registrar	1.4 (1.0–1.8)		2.5 (2.2–2.8)	
Junior doctor	1.4 (0.7–2.1)		2.8 (1.8–3.7)	
Other	1.6 (0.8–2.4)		3.6 (2.6–4.6)	
**Department**		0.19		<0.001
Medical specialities	1.7 (1.4–2.0)		2.7 (2.6–2.9)	
Surgical specialities	1.8 (1.5–2.1)		2.9 (2.7–3.1)	
Anaesthesia or intensive care medicine	1.7 (1.5–1.9)		2.8 (2.5–3.1)	
Psychiatry or addiction medicine	2.2 (1.8–2.6)		3.5 (3.2–3.8)	
Other	2.6 (1.6–3.7)		2.8 (2.3–3.3)	
**Manager**				0.08
No	1.8 (1.6–2.0)	0.36	2.8 (2.7–3.0)	
Yes	2.0 (1.6–2.5)		3.1 (2.8–3.5)	
**Directly involved in treatment or care of COVID-19 patients**		0.006		0.09
No	1.7 (1.5–1.9)		2.8 (2.7–3.0)	
Yes	2.1 (1.9–2.4)		3.0 (2.8–3.2)	
**Redeployed or given other tasks due to COVID-19**		0.02		<0.001
No	1.7 (1.6–1.9)		2.8 (2.6–2.9)	
Yes	2.2 (1.9–2.6)		3.4 (3.1–3.7)	

^a^ Score which roughly corresponds to the number of priority-setting dilemmas experienced in the previous two weeks.

^b^ On a scale from 0 (‘none’) to 10 (‘worst possible’).

**Figure 1.**
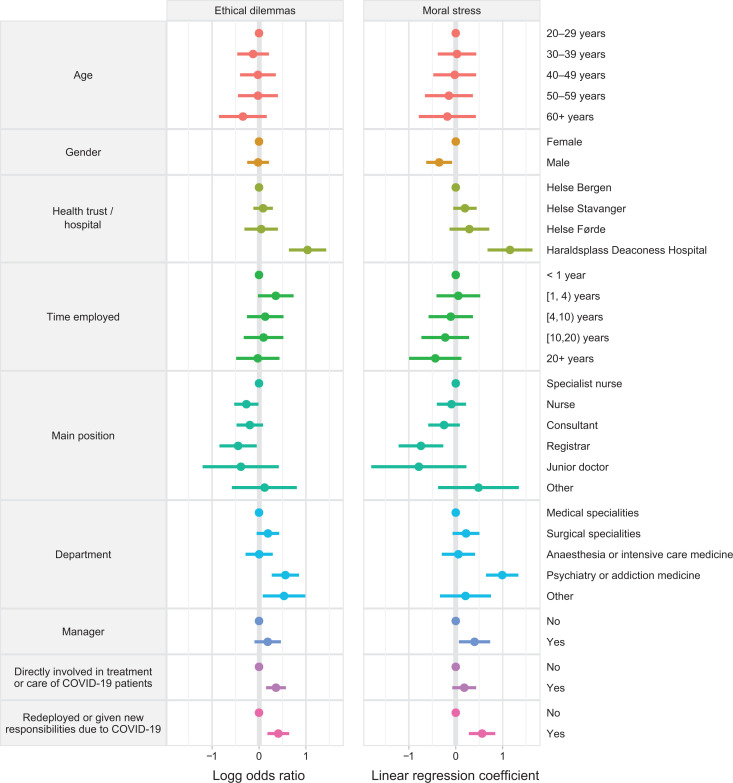
Multiple regression models for the association between the frequency of experienced priority-setting dilemmas due to resource scarcity or the level of moral distress in the previous two weeks and various demographic and occupational characteristics (*n* = 1,606). Regression coefficients with 95% confidence intervals are shown. For both models, coefficients > 0 are worse, i.e. they indicate higher frequencies of dilemmas or higher moral distress scores (compare to reference categories).

Figure 1 displays the frequency of priority-setting dilemmas and moral distress using proportional odds and linear multiple regression models.

These results are presented in more detail in Supplemental Table A. Individuals who had been directly involved in treatment or care of COVID-19 patients, worked at HDS or had been redeployed or given other tasks in response to COVID-19 were predicted to report a higher number of priority-setting dilemmas. Individuals working in psychiatry and addiction medicine or ‘other’ department also experienced priority-setting dilemmas more frequently than someone working within the medical department (reference group).

Participants were also asked about the most severe consequences they had seen as a result of resource scarcity during the past 2 weeks. Overall, 59% reported impacts of limited resources. Changes in treatment or missing treatment (46%) was the most common consequence, followed by accidents without serious consequences (7%) and temporary loss of function (4%). Few reported severe consequences, such as permanent loss of function (0.8%), life-threatening situations (1.4%) or death (0.2)

### Moral distress

Overall, the mean moral distress level on the 0–10 scale the past two weeks was 2.9 (95% CI 2.8–3.0). In adjusted analyses, a health worker who had been redeployed, worked at HDS, in psychiatry and addiction medicine or with management responsibility reported higher moral distress (Figure 1, Supplemental Table A). Male health workers reported significantly lower moral distress. There was a significant interaction effect between department and being directly involved and between department and being redeployed. Among those directly involved with COVID-19 patients or redeployed, a worker in the surgical specialities reported more moral distress than someone in the medical department, while this was not seen among those who were not directly involved or redeployed. Further, managers reported higher moral distress than non-managers only among those who were not directly involved.

Those employed at HDS reported higher moral distress, independent of whether they had been involved in COVID-19 care or whether they had been redeployed.

### Support mechanisms

**Figure 2.**
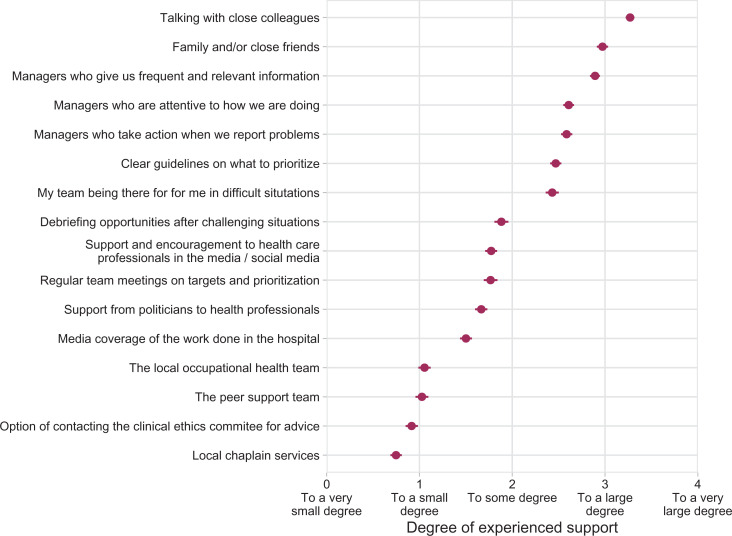
Mean scores with 95% confidence intervals for the degree of experienced support from various support options (*n* = 1606).

A range of mechanism was seen as supportive during the COVID-19 outbreak (Figure 2). Mechanisms that were built on existing collegial and managerial structures and routines, attention from colleagues, managers, family and friends, access to information and guidance were seen as supportive or very supportive.

**Figure 3.**
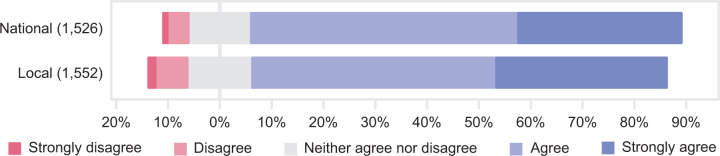
Agreement with national and local prioritisation guidelines (*n* = 1566). The number of respondents to each question is listed in parentheses.

The health workers who expressed an opinion mostly agreed with the COVID-19 priority-setting guidelines. For the national and local guidelines, respectively, 83% and 80% agreed or strongly agreed, and only 5% and 7% disagreed or strongly disagreed (Figure 3).

## Discussion

The aim of this study was to describe priority-setting dilemmas, moral distress and support experienced by nurses and physicians in the early phase of the COVID-19 pandemic in the Western part of Norway. Findings reveal that among respondents from all specialities, two thirds had experienced priority-setting dilemmas the previous 2 weeks. Despite predictions of potential severe resource scarcity, few reported severe adverse consequences. Moral distress levels were generally low, but those redeployed, with management responsibility or working in psychiatry and addiction medicine reported higher levels. Support mechanisms such as discussions with colleagues, family and friends, attentive managers and priority-setting guidelines were seen as most helpful.

### New demands and uncovered needs

The proportion of participants experiencing priority-setting dilemmas (67%) the previous two weeks was high. This finding might be surprising, given that the survey was conducted after the peak of COVID-19 cases and the limit in available ICU beds and general admissions was never reached. The high frequency of priority-setting dilemmas can be explained by COVID-19 specific challenges and underlying structural factors. Healthcare workers who were directly involved in the treatment of COVID-19 patients or had been redeployed experienced more priority-setting dilemmas, which may be explained by the burden of decision-making in high-risk settings and managing new and additional tasks, sometimes with little training. Studies of priority-setting dilemmas in later phases of the response, as well as a in non-COVID-19 setting, are necessary to explore these findings further.

Healthcare workers in psychiatry and addiction medicine reported a higher number of priority-setting dilemmas. Due to redeployment and reduced provision of services following reallocation of resources from mental healthcare to somatic healthcare and infection control measures, health workers could not physically meet and see their patients. These potentially vulnerable patients were also hindered in participating in social and supportive activities during lockdown. New reports show increases in calls from people with suicidal behaviour and loneliness.^31^ Being prevented from offering potentially beneficial services to a worse-off population during COVID-19 might also explain why this group reported higher levels of moral distress. On the other hand, frequent priority-setting dilemmas within psychiatry and addition might also reflect well-known existing resource scarcity prior to COVID-19.

### At risk of moral distress

Our findings on priority-setting dilemmas and moral distress relate to other studies describing correlations between experienced resource scarcity and moral distress across professions, specialisations and contexts.^15,32,33^ Experiences and levels of moral distress depend on external constraints, such as inadequate communication among team members, internal constraints, such as a lack of empowerment or self-doubt, and the clinical situations.^34,35^ The direct and indirect effects of the COVID-19 responses may influence these factors in multiple ways.^36^ Our survey revealed that those redeployed showed higher levels of moral distress, which is in line with findings that novices report higher moral distress.^15^ It may be associated with lack of understanding of the situation or treatment alternatives, hierarchies or insufficient training.

Dunn et al.^37^ discuss ethical issues associated with changes to staff allocation processes in the face of COVID-19, and points at how health professionals are insecure and worry about whether and how they themselves will be allocated to high-risk clinical roles. Our study provides solid empirical evidence for the increased moral distress among redeployed employees, even in a setting with low COVID-19 patient caseload and adequate resources. Similarly, those with management responsibilities experience higher levels of moral distress, and in particular managers who were not directly involved in COVID-19 care. Increased responsibility as well as anticipation of unpredictable situations are known to activate stress.^38,39^ Female participants reported higher levels of moral distress. Gender differences require further study, but they are consistent with findings from other studies.^40,41^

### Information and support from peers and managers matter most

Support from colleagues, family, friends and managers were found most helpful for healthcare professionals in the early phase of the pandemic. Building on these findings, ensuring that healthcare staff have time and possibility to share experiences with colleagues seems to be crucial in demanding situations, such as COVID-19. Making use of available support has previously been shown to be more likely if staff perceives the working environment to be supportive.^42^ Given that priority-setting dilemmas and moral distress varied also among those not directly involved in COVID-19 care, a supportive working environment is important for everyone and should build on existing structures.

Having a manager that provided frequent and relevant information, who were attentive to how the healthcare workers were doing and who would take action when they reported problems were experienced as supportive. This is in line with perspectives of how leadership is pivotal in enabling development and preservation of supportive and empathic healthcare organisations.^43^ Supportive leadership is of high importance, but even more so in situations with elevated demands, high level of uncertainty and increased needs for resilience, such during a pandemic.^44^ Studies from other contexts where work pressure is high have found that supportive leadership correlates with employee resilience.^45^

Well-functioning support mechanisms can protect against moral distress.^46^ Support from existing clinical and managerial structures was found important across participants. While levels of moral distress generally were low, the increased levels of moral distress among some subgroups may relate to COVID-19 related changes or underlying factors rather than lack of support mechanisms.

Pandemic-specific support activities, such as peer support teams, were new and may not have been fully accessible when the study was conducted. This can explain why these were not seen as more helpful. Pandemic-specific support activities may also target those directly working with COVID-19, whereas our findings indicate that also other employees are at risk. Yet, healthcare workers may also compensate and adjust to new situations amid crises, whereas reactions and consequences such as burn-out symptoms and even PTSD have been shown to occur later.^47^ Support for employees who experience adverse consequences in the aftermath of the pandemic is of key importance.^44^ Based on the findings in the present study, we recommend that efforts to address and prevent moral distress should be based on strengthening institutional structures and values rather than implementing resource-demanding expert consultations and ad hoc initiatives. Furthermore, managers and caring colleagues should be supported in efforts to identify employees who struggle at work. This may prevent moral distress and job resignations.^48^

### Priority-setting guidelines are important

Clear guidelines on what to prioritise were also seen as supportive. We believe this is linked to the finding that 4 out of 5 responders agreed with national and local COVID-19 guidelines (Figure 3), and only about 1/20 disagreed. Researchers and clinicians have argued for clear triage- and priority-setting criteria and regulations in treatment of COVID-19 patients and in the pandemic response, and what these should look like, have been debated.^2,3^ Studies show the challenge of getting clinicians to use guidelines if they disagree or find them unsuitable for their clinical context.^49^ The high level of agreement on guidelines among our respondents is relevant in discussions of whom (criteria for priority-setting) and how (priority-setting processes) to prioritise in a time of COVID-19-related scarcity.

Norway has a long and comprehensive tradition for institutionalised priority setting and related processes, in which the COVID-19 guidelines were developed and then implemented.^22^ The COVID-19 guidelines were sent out for comments and were changed substantially in response to various inputs. Triage recommendations and COVID-19 guidelines have been seen as controversial and have led to heated debates in many countries. Our results show that the Norwegian COVID-19 recommendations were widely accepted among healthcare providers. The guidelines are in accordance with the *general* priority-setting guidelines and provide detailed recommendations on issues like limiting admission of nursing-home patients to hospitals if they tested positive for COVID-19.^22^ The existing trust and credibility of the health authorities and the open and transparent processes may have facilitated support.^50^ These findings suggest that the development of clinical priority-setting guidelines for handling situations such as the COVID-19 pandemic should be a priority, since it supports frontline workers. This finding is likely to be relevant beyond the Norwegian setting, even if legitimacy and perceived relevance of clinical guidelines are likely to depend on contextual factors, established mechanisms for involvement and available resources.

### Strengths and limitations

Reports and stories from epicentres are valuable.^2^ Our study provides an example from a different COVID-19 setting and illustrates the importance and need for empirical ethics studies during a pandemic. Two important strengths of the present study should be noted; we were able to explore how experiences varied across hospitals, departments and roles, and the study revealed what forms of support healthcare workers themselves found useful. The duty to support and care for the healthcare workers during COVID-19 are being debated, and these discussions should be informed by our empirical findings.

This cross-sectional survey displays the experienced priority-setting dilemmas and moral distress in April–May 2020 in Norway, and the study findings may not be generalizable across contexts and time. Longitudinal studies of the priority-setting dilemmas, moral distress and support are needed to understand the causal effects of COVID-19. The response rate was quite low (18%), which may be due to limited time or access to computers or email at work. Healthcare workers more affected or interested in COVID-19 responses might have been more likely to participate, which may have biased our results. However, the distribution of background variables was similar in our sample as seen in the source population, which is reassuring. Another potential limitation is the moral distress scale, which was used as a simplified proxy to capture the complex experience of moral distress. However, it is user-friendly, and we argue that it can be useful until better measurements of moral distress are developed for use across professions, hospital units and different contexts.

## Conclusion

Our study describes a portrait of healthcare professionals’ experiences following initial responses to the COVID-19 outbreak in the Norwegian health system. Priority-setting dilemmas were common, but levels of moral distress were low. Further studies on the experiences of healthcare workers who are both directly and indirectly affected by the COVID-19 response is needed to prepare for the next phases of the pandemic and future health emergencies. Support – in terms of resources and protective working environments – is key to prepare healthcare workers to do their best in ethically challenging situations related to COVID-19 and in other situations.

## Supplemental material

Supplemental Material, sj-pdf-1-nej-10.1177_0969733020981748 - Priority-setting dilemmas, moral distress and support experienced by nurses and physicians in the early phase of the COVID-19 pandemic in Norway Click here for additional data file. Supplemental Material, sj-pdf-1-nej-10.1177_0969733020981748 for Priority-setting dilemmas, moral distress and support experienced by nurses and physicians in the early phase of the COVID-19 pandemic in Norway by Ingrid Miljeteig, Ingeborg Forthun, Karl Ove Hufthammer, Inger Elise Engelund, Elisabeth Schanche, Margrethe Schaufel and Kristine Husøy Onarheim in Nursing Ethics

## Note

i. Numbers from Norwegian Intensive Care Registry, https://helse-bergen.no/norsk-intensivregister-nir
