# Supplementary material for: Priority-setting dilemmas, moral distress and support experienced by nurses and physicians in the early phase of the COVID-19 pandemic in Norway
Source: Nurs Ethics. 2021 Jan 12;28(1):66–81. doi: 10.1177/0969733020981748 (PMC7879232; doi:10.1177/0969733020981748)
Supplement: Supplemental Material, sj-pdf-1-nej-10.1177_0969733020981748 - Priority-setting dilemmas, moral distress and support experienced by nurses and physicians in the early phase of the COVID-19 pandemic in Norway [file sj-pdf-1-nej-10.1177_0969733020981748.pdf]

	ETHICAL DILEMMAS			MORAL DISTRESS		
	Log odds ratio (95% CI)			Linear regression coefficient (95% CI)		
	**Crude**	**Adjusted[1]**	**Adjusted[2]**	**Crude**	**Adjusted[1]**	**Adjusted[2]**
**Age**						
20–29 years	0.07<br>(−0.21; 0.36)	−0.02<br>(−0.37; 0.33)	0.02<br>(−0.36; 0.40)	0.28<br>(−0.07; 0.64)	0.03<br>(−0.40–0.46)	0.02<br>(−0.43; 0.48)
30–39 years	−0.12<br>(−0.36; 0.12)	−0.17<br>(−0.44; 0.09)	−0.10<br>(−0.38; 0.18)	0.03<br>(−0.27; 0.32)	−0.09<br>(−0.41–0.24)	0.05<br>(−0.29; 0.38)
40–49 years	Ref.	Ref.	Ref.	Ref.	Ref.	Ref.
50–59 years	−0.05<br>(−0.31; 0.20)	0.06<br>(−0.22; 0.34)	0.00<br>(−0.28; 0.28)	−0.16<br>(−0.47; 0.16)	−0.04<br>(−0.38–0.31)	−0.12<br>(−0.46; 0.21)
60+ years	−0.38<br>(−0.74; −0.03)	−0.29<br>(−0.67; 0.10)	−0.32<br>(−0.71; 0.07)	−0.28<br>(−0.71; 0.14)	−0.10<br>(−0.56–0.36)	−0.16<br>(−0.61; 0.30)
**Sex**						
Female	*Ref.*	*Ref.*	*Ref.*	*Ref.*	*Ref.*	*Ref.*
Male	0.00<br>(−0.21; 0.21)	−0.01<br>(−0.22; 0.21)	−0.02<br>(−0.25; 0.21)	−0.39<br>(−0.65; −0.13)	−0.40<br>(−0.67; −0.14)	−0.35<br>(−0.63; −0.08)
**Time employed**						
< 1 year	−0.13<br>(−0.47; 0.20)	−0.18<br>(−0.56; 0.21)	−0.10<br>(−0.52; 0.32)	0.14<br>(−0.28; 0 .56)	0.04<br>(−0.43; 0.51)	0.22<br>(−0.28; 0.73)
[1–4) years	0.24<br>(−0.04; 0.52)	0.26<br>(−0.06; 0.58)	0.26<br>(−0.08; 0.60)	0.26<br>(−0.08; 0.61)	0.23<br>(−0.16; 0.63)	0.28<br>(−0.13; 0.68)
[4–10) years	−0.01<br>(−0.27; 0.24)	0.02<br>(−0.25; 0.30)	0.04<br>(−0.25; 0.32)	0.09<br>(−0.22; 0.40)	0.10<br>(−0.24; 0.43)	0.12<br>(−0.22; 0.45)
[10–20) years	Ref.	Ref.	Ref.	Ref.	Ref.	Ref.
20+ years	−0.17<br>(−0.42; 0.08)	−0.17<br>(−0.46; 0.11)	−0.12<br>(−0.42; 0.17)	−0.27<br>(−0.57; 0.04)	−0.28<br>(−0.62; 0.07)	−0.21<br>(−0.56; 0.14)
**Health trust / hospital**						
Helse Bergen	*Ref.*	*Ref.*	*Ref.*	*Ref.*	*Ref.*	*Ref.*
Helse Stavanger	0.22<br>(0.02; 0.42)	0.24<br>(0.04; 0.44)	0.09<br>(−0.12; 0.30)	0.40<br>(0.15; 0.64)	0.39<br>(0.14; 0.64)	0.20<br>(−0.05; 0.45)
Helse Førde	0.04<br>(−0.31; 0.39)	0.04<br>(−0.32; 0.39)	0.05<br>(−0.31; 0.40)	0.25<br>(−0.17; 0.68)	0.31<br>(−0.12; 0.74)	0.29<br>(−0.13; 0.71)
Haraldsplass Deaconess Hospital	1.11<br>(0.73; 1.50)	1.10<br>(0.72; 1.49)	1.03<br>(0.64; 1.43)	1.22<br>(0.74; 1.70)	1.19<br>(0.71; 1.67)	1.16<br>(0.68; 1.64)

**Main position**						
Specialist nurse	*Ref.*	*Ref.*	*Ref.*	*Ref.*	*Ref.*	*Ref.*
Nurse	−0.14<br>(−0.36; 0.07)	−0.27<br>(−0.51; −0.03)	−0.27<br>(−0.53; −0.01)	0.08<br>(−0.19; 0.35)	−0.13<br>(−0.43; 0.17)	−0.09<br>(−0.40; 0.22)
Consultant	−0.18<br>(−0.43; 0.08)	−0.20<br>(−0.47; 0.07)	−0.20<br>(−0.48; 0.09)	−0.34<br>(−0.64; −0.03)	−0.24<br>(−0.57; 0.08)	−0.25<br>(−0.59; 0.09)
Registrar	−0.32<br>(−0.64; 0.00)	−0.42<br>(−0.80; −0.03)	−0.44<br>(−0.84; −0.05)	−0.51<br>(−0.92; −0.10)	−0.77<br>(−1.24; −0.30)	−0.74<br>(−1.22; −0.27)
Junior doctor	−0.09<br>(−0.83; 0.66)	−0.21<br>(−1.02; 0.59)	−0.39<br>(−1.19; 0.42)	−0.20<br>(−1.16; 0.76)	−0.59<br>(−1.61; 0.43)	−0.79<br>(−1.80; 0.23)
Other	0.13<br>(−0.53; 0.79)	0.21<br>(−0.45; 0.87)	0.12<br>(−0.57; 0.81)	0.58<br>(−0.26; 1.42)	0.70<br>(−0.13; 1.54)	0.48<br>(−0.38; 1.35)
**Department**						
Medical specialities	*Ref.*	*Ref.*	*Ref.*	*Ref.*	*Ref.*	*Ref.*
Surgical specialities	0.11<br>(−0.12; 0.34)	0.12<br>(−0.12; 0.35)	0.19<br>(−0.05; 0.43)	0.17<br>(−0.12; 0.45)	0.18<br>(−0.10; 0.47)	0.22<br>(−0.07; 0.51)
Anaesthesia or intensive care medicine	0.20<br>(−0.06; 0.46)	0.19<br>(−0.08; 0.46)	0.00<br>(−0.29; 0.29)	0.09<br>(−0.23; 0.42)	0.15<br>(−0.17; 0.48)	0.06<br>(−0.30; 0.41)
Psychiatry or addiction medicine	0.38<br>(0.11; 0.65)	0.45<br>(0.17; 0.72)	0.56<br>(0.27; 0.85)	0.81<br>(0.48; 1.15)	0.89<br>(0.55; 1.22)	0.99<br>(0.65; 1.34)
Other	0.44<br>(−0.01; 0.88)	0.46<br>(0.01; 0.90)	0.53<br>(0.08; 0.99)	0.07<br>(−0.48; 0.62)	0.12<br>(−0.43; 0.66)	0.21<br>(−0.34; 0.75)
**Manager**						
No	*Ref.*	*Ref.*	*Ref.*	*Ref.*	*Ref.*	*Ref.*
Yes	0.13<br>(−0.12−0.39)	0.16<br>(−0.11; 0.42)	0.19<br>(−0.10; 0.47)	0.30<br>(−0.01; 0.61)	0.42<br>(0.10; 0.75)	0.40<br>(0.07; 0.73)
**Directly involved in treatment or care of COVID-19 patients**						
No	*Ref.*	*Ref.*	*Ref.*	*Ref.*	*Ref.*	*Ref.*
Yes	0.39<br>(0.21; 0.58)	0.30<br>(0.11; 0.50)	0.36<br>(0.15; 0.57)	0.20<br>(−0.03; 0.43)	0.08<br>(−0.16; 0.31)	0.18<br>(−0.07; 0.44)

**Redeployed or given new responsibilities due to COVID-19**						
No	*Ref.*	*Ref.*	*Ref.*	*Ref.*	*Ref.*	*Ref.*
Yes	0.46 (0.24; 0.68)	0.40 (0.17; 0.62)	0.41 (0.18; 0.64)	0.63 (0.36; 0.90)	0.51 (0.23; 0.79)	0.56 (0.28; 0.84)

Supplemental Table A: Univariate and multivariate analyses for predictors of frequency of priority setting dilemmas and moral distress the previous two weeks (*n* = 1,606).

[1] Adjusted for sex, age, years of experience and hospital trust.
[2] Mutually adjustment for all variables included in table.
